# White matter microstructural organization and gait stability in older adults

**DOI:** 10.3389/fnagi.2014.00104

**Published:** 2014-06-10

**Authors:** Sjoerd M. Bruijn, Annouchka Van Impe, Jacques Duysens, Stephan P. Swinnen

**Affiliations:** ^1^Motor Control Laboratory, Movement Control and Neuroplasticity Research Group, Department of Kinesiology, KU LeuvenLeuven, Belgium; ^2^Faculty of Human Movement Sciences, Research Institute MOVE, VU UniversityAmsterdam, Netherlands; ^3^Department of Orthopedics, First Affiliated Hospital of Fujian Medical UniversityFuzhou, China; ^4^Department of Research, Development and Education, Sint MaartenskliniekNijmegen, Netherlands; ^5^Leuven Research Institute for Neuroscience & DiseaseLeuven, Belgium

**Keywords:** gait stability, ageing, DTI, white matter integrity, extrapolated center of mass, maximum Lyapunov exponent, step width

## Abstract

Understanding age-related decline in gait stability and the role of alterations in brain structure is crucial. Here, we studied the relationship between white matter microstructural organization using Diffusion Tensor Imaging (DTI) and advanced gait stability measures in 15 healthy young adults (range 18–30 years) and 25 healthy older adults (range 62–82 years). Among the different gait stability measures, only stride time and the maximum Lyapunov exponent (which quantifies how well participants are able to attenuate small perturbations) were found to decline with age. White matter microstructural organization (FA) was lower throughout the brain in older adults. We found a strong correlation between FA in the left anterior thalamic radiation and left corticospinal tract on the one hand, and step width and safety margin (indicative of how close participants are to falling over) on the other. These findings suggest that white matter FA in tracts connecting subcortical and prefrontal areas is associated with the implementation of an effective stabilization strategy during gait.

## Introduction

Since gait is one of the most common tasks in daily life, and since falls often occur during gait (Rubenstein, 2006), understanding the decline in gait stability with ageing is a critical scientific endeavor.

Decreased stability in older adults may be due to losses in force production (Pijnappels et al., 2008), proprioception (Goble et al., 2009), vision (Hallemans et al., 2010), and vestibular function (Fife and Baloh, 1993). In addition to these peripheral changes, gait stability may be compromised because of an age-related decline in both gray (Good et al., 2001; Smith et al., 2007; Kalpouzos et al., 2009) and white matter microstructure (Nusbaum et al., 2001; Sullivan and Pfefferbaum, 2006, 2007).

While the influence of peripheral factors on gait stability has received ample attention in the literature (Dingwell et al., 2007; Kang and Dingwell, 2008; Pijnappels et al., 2008), research on the effect of central factors such as age related decrements in gray and white matter structure has been limited (Srikanth et al., 2009; Koo et al., 2012; Zheng et al., 2012).

The spinal and supraspinal mechanisms of gait have been studied intensively in animal and human systems (Duysens and van de Crommert, 1998), and recent advances in medical imaging have shifted the emphasis to the role of cortical and subcortical structures (Fukuyama et al., 1997; Jahn et al., 2004, 2008; Hanakawa, 2006; Bakker et al., 2007; Harada et al., 2009; Wang et al., 2008; Gwin et al., 2010, 2011; Snijders et al., 2011). However, most of these studies used functional Magnetic Resonance Imaging (fMRI) (Jahn et al., 2004, 2008; Hanakawa, 2006; Bakker et al., 2007; Wang et al., 2008; Snijders et al., 2011), which does not allow for the investigation of actual locomotion due to limited bore space and restricted head movement. Nevertheless, by combining fMRI and PET la Fougere et al. (2010) were able to show that real and imagined locomotion recruit a similar brain network comprising the frontal, primary and supplementary motor, and somatosensory cortices, cerebellum, pontomesencephalic tegmentum, parahippocampal, fusiform and occipital gyri, and basal ganglia. Locomotor-related activation was found in similar brain regions in several other studies using various paradigms including ankle dorsiflexion, imagery of locomotion, and EEG or near infrared spectroscopy during locomotion (Fukuyama et al., 1997; Miyai et al., 2001; Dobkin et al., 2004; Jahn et al., 2004, 2008; Hanakawa, 2006; Bakker et al., 2007; Wang et al., 2008; Harada et al., 2009; Gwin et al., 2010, 2011; Snijders et al., 2011).

How the aforementioned brain areas functionally contribute to gait needs to be examined in more detail. For instance, it has been suggested that the subcortical areas are important in generating rhythms (Jahn et al., 2008). Cortical areas have been suggested to play an important role in integration of sensory information and maintenance of stability during gait (la Fougere et al., 2010). The idea of cortical areas being important for maintenance of stability during gait is also supported by dual-tasking studies, showing decreases in gait stability when cognitive tasks are performed (Woollacott and Shumway-Cook, 2002).

While the brain networks required for *basic locomotor rhythm generation* can be studied using fMRI (combined with imagined locomotion), this is not possible for maintenance of gait *stability*, because experimentally altering the stability demands in a scanner-based experiment is obviously challenging. An alternative approach is inspired by the observation that the brain is a highly interconnected structure that is critically dependent on the exchange of information between spatially remote regions for optimal function. This can be addressed indirectly by determining the structural integrity of white matter (tracts) and correlating these with stability measures. Using this approach, the relationship between gait, falls and white matter lesions (WML's, which can be identified on MRI scans as white matter hyperintensities) has been established in previous studies (Baloh et al., 2003; Starr et al., 2003; Srikanth et al., 2009, 2010; Zheng et al., 2012). Together, these studies have shown a significant relationship between severity of WML's and falls, gait, and balance. Only in few of these studies, however, the relationship between *location* of WML's and balance and/or fall metrics was assessed (For a review see: Zheng et al., 2011). The latter work revealed that WML's in periventricular regions are related to decline in gait (Srikanth et al., 2009, 2010), while balance may be related to deep frontal WML's (Blahak et al., 2009). Lastly, only one study reported that the presence of prefrontal periventricular WML's was related to fall risk (Blahak et al., 2009) and this is consistent with the idea that cortico-subcortical connections may be important in maintaining gait stability. Even though the categorization of WML's has revealed valuable information, it provides less insight into specific tracts that might be involved.

Diffusion tensor imaging (DTI) combined with white matter tractography may be a better means of studying the relationship between location and status of white matter microstructural organization and behavior, as it has superior spatial resolution. Moreover, it can be used to assess the microstructural integrity of white matter in the absence of white matter lesions. Several recent studies have used this approach to study the relationship between gait, falls, and white matter (Bhadelia et al., 2009; de Laat et al., 2011; Koo et al., 2012). For instance, a recent study by Koo et al. (2012) reported relationships between fall risk and white matter metrics in a cohort of 125 homebound older adults. They observed significant correlations between increased fall risk and reduced *FA* in the medial frontal and parieto-subcortical pathways, genu and splenium of corpus callosum, posterior cingulum, prefrontal and orbitofrontal pathways, and frontoparietal and frontotemporal pathways. While these are interesting findings, probability of falling depends on many factors and therefore it is necessary to unravel the important contributors. A primary candidate for fall risk is gait instability. Therefore the present study has focused on this element in relation to white matter microstructural organization. For this purpose, we relied on a sensitive measure of stability (safety margin and short-term maximum Lyapunov exponent). Such measures were shown to be linked to the probability of falling (Bruijn et al., 2012; Toebes et al., 2012).

In the current study, we used DTI to quantify the microstructural organization of brain white matter, combined with state-of-the-art gait stability measures (Bruijn et al., 2013) to test the hypothesis that the microstructural organization of white matter tracts conveying information between cortical and subcortical structures, would be predictive of gait stability.

In a simple model of human walking (i.e., a 3D passive dynamic walking model) only frontal plane movements need to be controlled to prevent falling (Kuo, 1999). Moreover, it has been reported that mediolateral (ML) visual (O'connor and Kuo, 2009) and mechanical (McAndrew et al., 2010, 2011) perturbations affect human gait more than anterior-posterior perturbations. Accordingly, we focused on mediolateral stability of gait.

## Methods

### Participants

Fifteen healthy young adults [8 female, *M* = 22.4 years, range 18–30 years, length 1.69 ± 0.09 m (*M* ± *SD*), weight 76.3 ± 13.1 kg (*M* ± *SD*)] and twenty-five healthy older adults [10 females, *M* = 70.9 years, range 62–82 years, length 1.76 ± 0.08 m (*M* ± *SD*), weight 67.4 ± 10.3 kg (*M* ± *SD*)] participated in the study. All participants had normal or corrected to normal vision and were right-handed, as assessed by the Edinburgh Handedness Inventory (Oldfield, 1971). The Montreal Cognitive Assessment Test was used to determine general cognitive function in the older adults. All scored within normal limits (score ≥26 out of 30). Before participating in the experiment, participants were screened for possible contra-indications and medication use, using a standard pre-scanning questionnaire. None of the participants had any self-reported complaints (either neurological or orthopedic) that would interfere with normal walking. They were informed about the experimental procedures and provided written informed consent. The study was approved by the local Ethics Committee of KU Leuven and was performed in accordance with the 1964 Declaration of Helsinki.

### Procedure: Gait assessment

For the assessment of gait stability we used treadmill walking, as it allows measuring multiple subsequent strides, a necessity for some stability measures. When entering the lab, participants were first outfitted with a cluster of 3 retro reflective markers, which was placed on the sacrum. This cluster was used for 3D movement analysis using a Vicon MX system (Oxford Metrics, Oxford, UK). After familiarization with the treadmill, data were recorded while the participants walked for 5 min at 1 m/s. 3D kinematics of the pelvis cluster marker were sampled at 100 samples/s, and 3D ground reaction forces and torques were sampled at 1000 samples/s using inbuilt treadmill sensors (custom made by Forcelink, Culemborg, The Netherlands), allowing for calculation of the center of pressure.

### Procedure: Scanning

Scanning was performed on a Philips 3T Achieva MRI scanner (Philips, Best, The Netherlands) with a 32-channel matrix head coil. First, a 3D MPRAGE high-resolution T1-weighted image (*TR* = “shortest,” *TE* = 4.60 ms, flip angle = 8, 230 sagittal slices each 1 mm thick, in plane resolution = 0.97 × 0.98, 384 × 384 matrix) was acquired to check for possible abnormalities in gray matter. Second, single shot spin-echo diffusion weighted images (*TR* = “shortest,” *TE* = 56 ms, 50 axial 2.2 mm slices with 0.29 mm gap, in plane resolution 2 × 1.96 mm, matrix 220 × 220) with diffusion sensitizing gradients applied along 75 non-collinear directions (*b*-value of 800 s/mm^2^) were acquired. In addition, one image with no diffusion weighting (b0) was acquired. Acquisition time of the MPRAGE scan was 6 min and 56 s, while that of the diffusion scan was 13 min and 16.4 s.

### Calculations

#### Gait data

***Preprocessing***. Gait events (time points at which the feet touched or left the support surface) were detected from the center of pressure (CoP) trajectories using a previously described algorithm (Roerdink et al., 2008). The location of the center of mass (CoM) was calculated from the CoP using a low pass filtering method (Hof, 2005). The positions of the three markers on the pelvis cluster were averaged to form one 3D time series of pelvis movement.

***Calculation of stability parameters***. Mediolateral gait perturbations elicit a reduction in stride time and an increase in step width, which have been suggested to stabilize gait (McAndrew et al., 2011; Hak et al., 2012). Thus, as a first set of gait parameters we calculated stride time (defined as the time difference between two consecutive ipsilateral heel strikes) and step width (defined as the ML distance between the CoP position during double stance).

ML foot placement is coordinated with ML trunk movement in order to stabilize gait in young and healthy older adults (Hurt et al., 2010). To quantify these effects, we calculated the ML amplitude of the CoM by taking the difference between maximum and minimum center of mass positions within a stride, and averaging over strides.

To express stability in terms of how close a participant is to falling over, we used the safety margin, as introduced by Hof et al. (2005, see also Supplementary Materials). This margin is the difference between the lateral edge of the base of support (i.e., the lateral CoP position), and the CoM, which is extended by taking CoM velocity into account. The mean distance during stance between the projections of the extrapolated CoM and CoP, was designated as the “safety margin” (Hof et al., 2005). A larger safety margin indicates that participants place their feet further away from their extrapolated CoM, it is thus harder for them to tip over.

While the above measures quantify how close a participant is to falling over, they do not capture the ability to attenuate the effects of small perturbations. A measure that is able to capture this ability is the short-term maximum Lyapunov exponent (λ_*S*_, see Supplementary Material). We calculated λ_*S*_ from the pelvis cluster ML velocity data (see Supplementary Materials). Higher values of λ_*S*_ indicate less local stability.

All calculations of gait variables were performed using custom Matlab (The MathWorks, Natick, MA) programs.

#### DTI processing

Diffusion data were pre-processed and analyzed using FSL version 4.1 (Smith et al., 2004). First, the b0 image of each participant was skull-stripped using the brain extraction tool, the data were corrected for participant motion and eddy-current induced geometrical distortions, and the diffusion sensitizing gradients (“bvecs”) were rotated to correct for motion. Using the FSL Diffusion Toolbox, the diffusion tensor model was fit to the data, from which fractional anisotropy (FA) images were calculated. High *FA* values are considered to reflect more coherent tissue structure.

We then used tract-based spatial statistics (TBSS) for voxel-based analyses of white matter structure across the whole brain. This method restricts analysis to an *FA* skeleton onto which the central white matter tract values for each participant are projected. It is a non-hypothesis driven approach that tests for correlations in all voxels that belong to the white matter skeleton. First, all participants' *FA* data were registered to a common space (the FA158 MNI space template) using a combination of affine and non-linear registration. A mean *FA* image was created, reduced to a skeleton and thresholded at *FA* > 0.25. Each subject's aligned *FA* data was then projected onto this skeleton and used in a voxelwise statistical analysis. Demeaned gait stability parameters were correlated against white matter integrity, with demeaned age included as a covariate of no interest (Randomize, 10,000 permutations). These correlations were performed for both groups separately because no significant correlations were expected for the group of younger participants, as inferred from our previous work (see for instance Van Impe et al., 2012). The theoretical rationale for an absence of significant correlations in younger adults is two-fold. Firstly, since younger adults will have a higher structural integrity of white matter than older adults (Nusbaum et al., 2001; Van Impe et al., 2012), it is less likely to be a limiting factor. Secondly, variation within groups of younger adults is usually smaller, and this lack of variance reduces the probability to obtain significant correlations. For all TBSS analyses, *P* < 0.05 was considered significant. This *P*-value was corrected by means of Threshold Free Cluster Enhancement (TFCE), which accounts for the multitude of correlation analyses across brain voxels by identifying clusters in the data (Smith and Nichols, 2009) was considered significant. The Johns Hopkins University (JHU) tractography atlas was used to identify significant voxels. When significant correlations were found for either of the groups using TBSS, the mean value of all significant voxels was extracted for both groups. Correlation coefficients of the two groups were then compared using the Fischer r-to-z transform. For the group in which a significant relationship was found, the *FA* value was plotted against the gait variable of interest, and the correlation coefficient between the *FA* value and the stability metric was calculated.

The significant cluster from our whole-brain *FA* correlation analysis for step width and safety margin occurred in a region of crossing fibers (see Figure 3). We therefore made use of a crossing fiber model (BEDPOSTX) to resolve which fiber population was driving the effect. The probabilistic diffusion model calculates probability distributions of fiber direction at each voxel in participant diffusion space, allowing for estimates of more than one fiber direction within a voxel (Behrens et al., 2003, 2007). Specifically, the contribution of primary (F1) and secondary (F2) fiber directions were calculated for each voxel. These scalar values were reassigned to obtain a consistent labeling of F1 and F2 within and across participants (Jbabdi et al., 2010). The resulting partial volume fractions (F1 and F2) were then used for TBSS, as described above.

### Statistical analyses

Gait variables were compared between groups using unpaired *t*-tests, after data normality was confirmed using Lilliefors test. *P* < 0.05 was considered significant throughout, and the Matlab statistics toolbox was used for all statistics other than TBSS.

## Results

### Age related changes in gait stability

**Figure 1:**
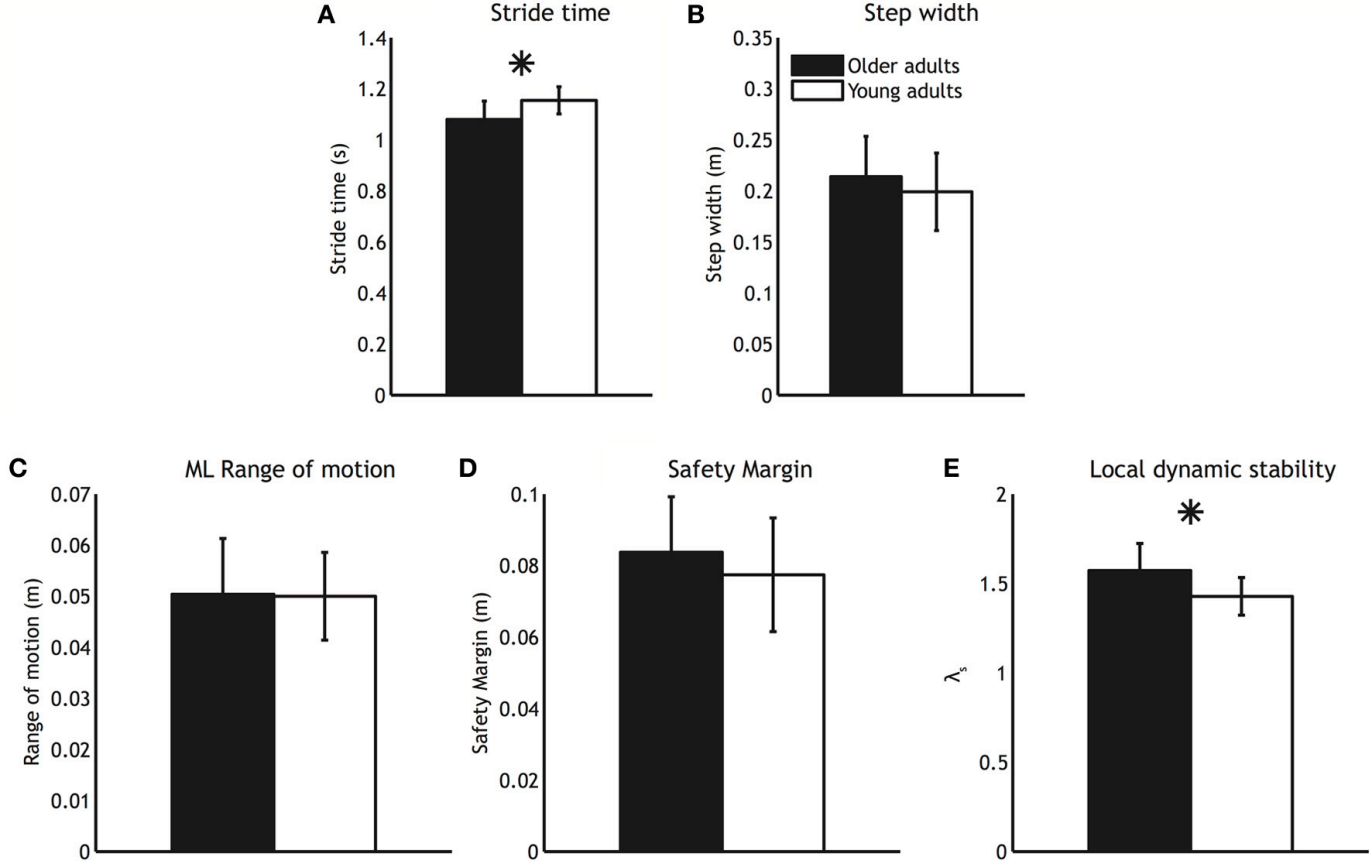
**Mean gait parameters for older adults (black bars) and younger adults (white bars). (A)** Stride time **(B)** Step width **(C)** Medio-lateral CoM range of motion **(D)** Safety margin and **(E)** Local dynamic stability. Error bars represent standard deviation, and the asterisk represents a significant group difference.

Compared to young adults, older adults walked with significantly shorter stride times (*P* < 0.01, see Figure 1A). Nevertheless, older adults did not differ with regard to their step widths (*P* = 0.25, see Figure 1B). Moreover, older adults did not differ from young adults in terms of ML CoM range of motion (*P* = 0.95, Figure 1C). Finally, there was no difference in the safety margin (*P* = 0.22, Figure 1D). There were high correlations between step width, ML CoM range of motion, and safety margin (see Table 1).

**Table 1:** **Means, standard deviations, and correlations between different gait parameters**.

**All participants**	**Mean (*SD*)**	**Step time**	**Step width**	**ML CoM range of motion**	**Safety margin**	**λ_*S*_**
Step time	1.11 (0.08) [s]	x				
Step width	0.21 (0.04) [m]	0.05	x			
ML CoM ROM	0.05 (0.01) [m]	**0.50**	**0.84**	x		
Safety margin	0.08 (0.02) [m]	−0.08	**0.98**	**0.73**	x	
λ_*S*_	1.52 (0.15) [-]	**−0.44**	0.08	−0.08	0.13	x
**YOUNGER ADULTS ONLY**						
Step time	1.15 (0.05) [s]	x				
Step width	0.20 (0.04) [m]	0.08	x			
ML CoM ROM	0.05 (0.01) [m]	0.50	**0.89**	x		
Safety margin	0.08 (0.02) [m]	−0.01	**0.99**	**0.83**	x	
λ_*S*_	1.43 (0.11) [-]	−0.50	0.27	0.10	0.30	x
**OLDER ADULTS ONLY**						
Step time	1.08 (0.07) [s]	x				
Step width	0.21 (0.04) [m]	0.22	x			
ML CoM ROM	0.05 (0.01) [m]	**0.63**	**0.85**	x		
Safety margin	0.08 (0.02) [m]	0.04	**0.97**	**0.70**	x	
λ_*S*_	1.57 (0.15) [-]	−0.21	−0.13	−0.18	−0.08	x

*Significant correlations are indicated in bold*.

While older adults were similar to young adults in terms of how close they were to falling over (i.e., had similar safety margins), they were less able to attenuate the effects of small perturbations, as evidenced by a higher λ_*S*_ (*P* < 0.01, see Figure 1E).

### Age related changes in white matter microstructural organization

**Figure 2:**
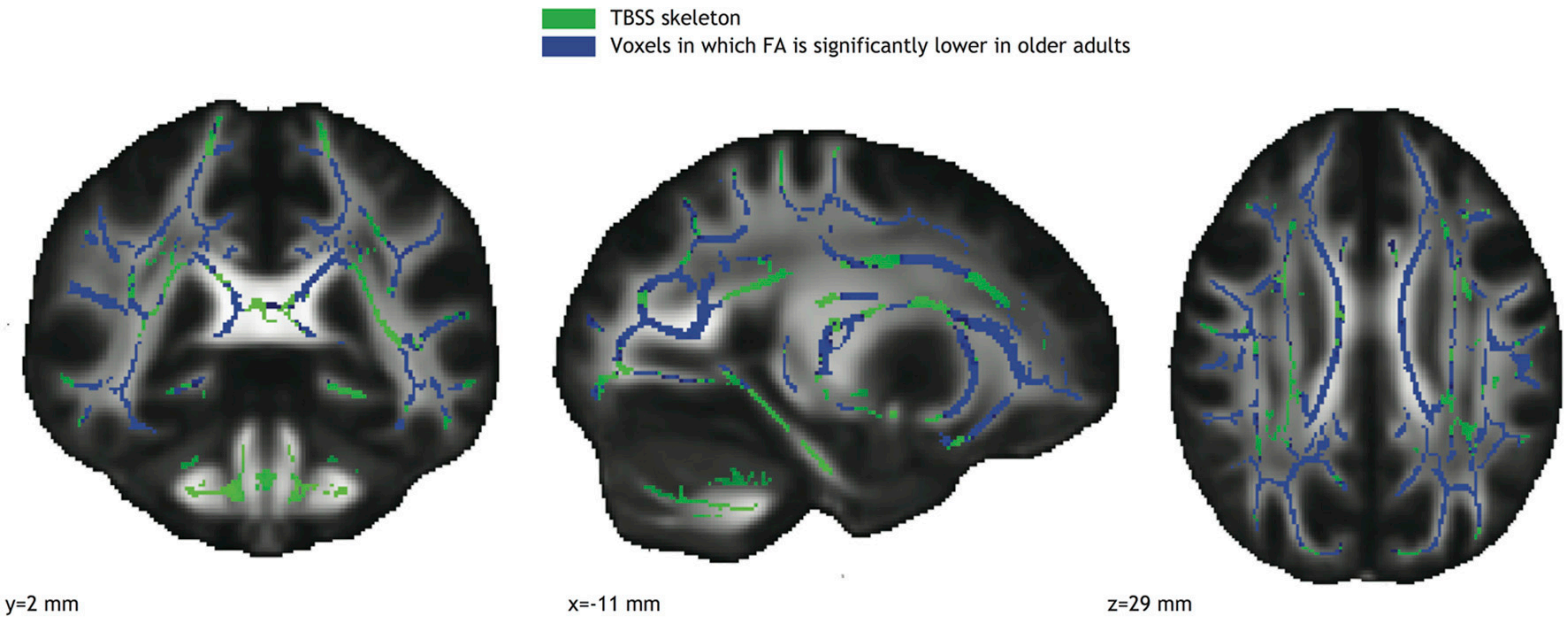
**Voxels with significantly reduced *FA* in the older adults group (blue; TFCE corrected) overlaid on the TBSS skeleton (green)**.

Widespread age related decreases in *FA* were detected in white matter pathways throughout the TBSS skeleton (Figure 2), including left and right corticospinal tracts, left and right anterior thalamic radiation, left and right superior and inferior longitudinal fasciculi, left and right inferior fronto-occipital fasciculi, and the corpus callosum.

### Correlations between white matter microstructural organization and gait stability

**Figure 3:**
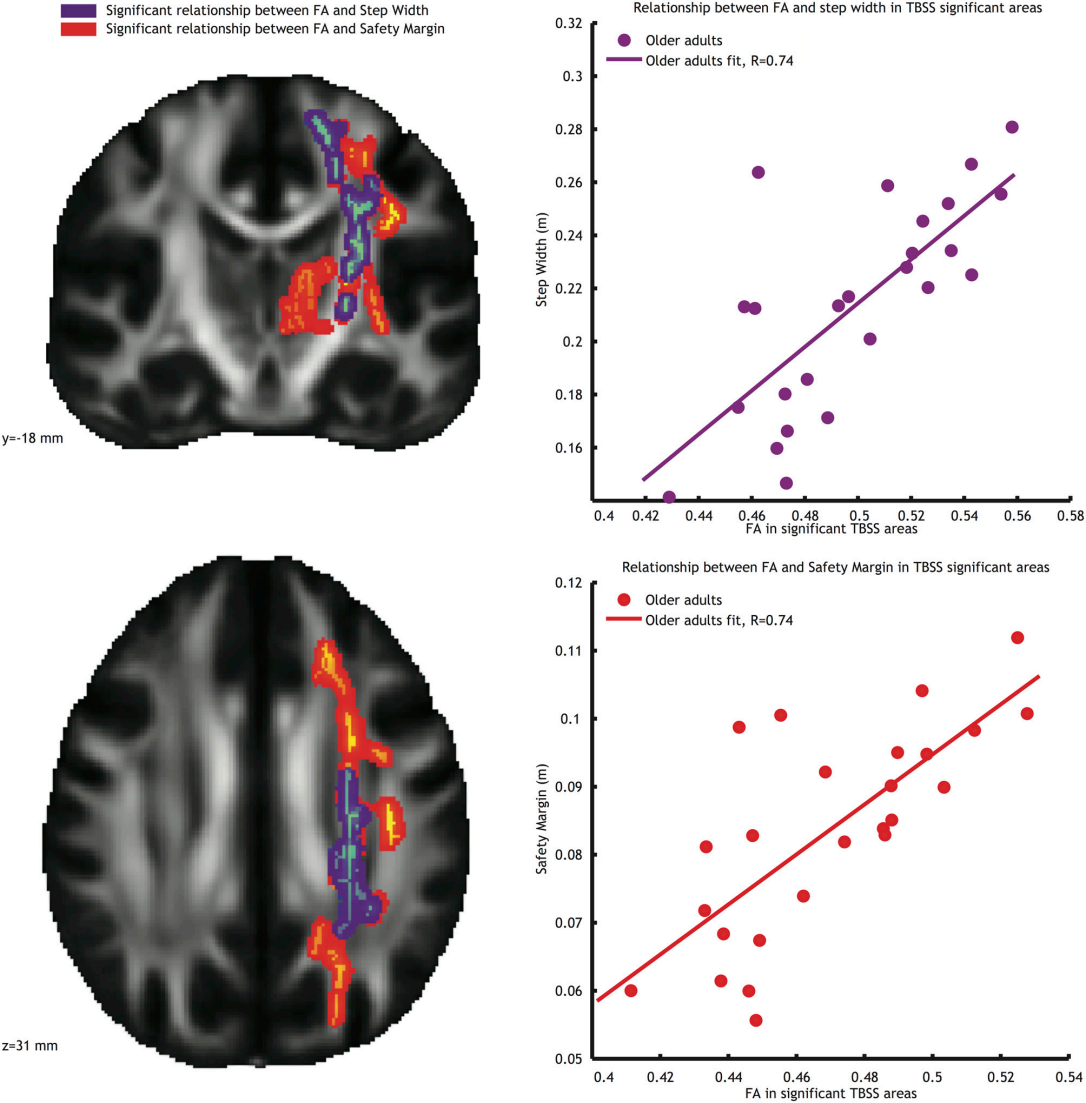
**Left panels**: Voxels showing significant correlations (*P* < 0.05, TFCE corrected) between *FA* and step width (blue-purple) and *FA* and safety margin (red) in the older adults group. **Right panels**: Correlations between mean *FA* value in the significant area and step width (top right panel), and between mean *FA* value in the significant area and safety margin (bottom right panel).

Significant correlations between *FA* values and gait parameters related to stability were seen in the older adults group (see Figure 3). Low *FA* values coincided with narrow step widths (*r* = 0.74, see Figure 3, blue-purple brain areas, and top right panel) and small safety margins (*r* = 0.74, see Figure 3, reddish brain areas and bottom right panel). For both these correlations, the significant voxels belonged to the left corticospinal tract, left anterior thalamic radiation, and left longitudinal fasciculus. Furthermore, the significant correlations observed between *FA* values and stability measures in the older adults group were reliably higher than those in the younger adults group (Fischer r-to-z transform, *P* < 0.01 for both step width and safety margin).

**Figure 4:**
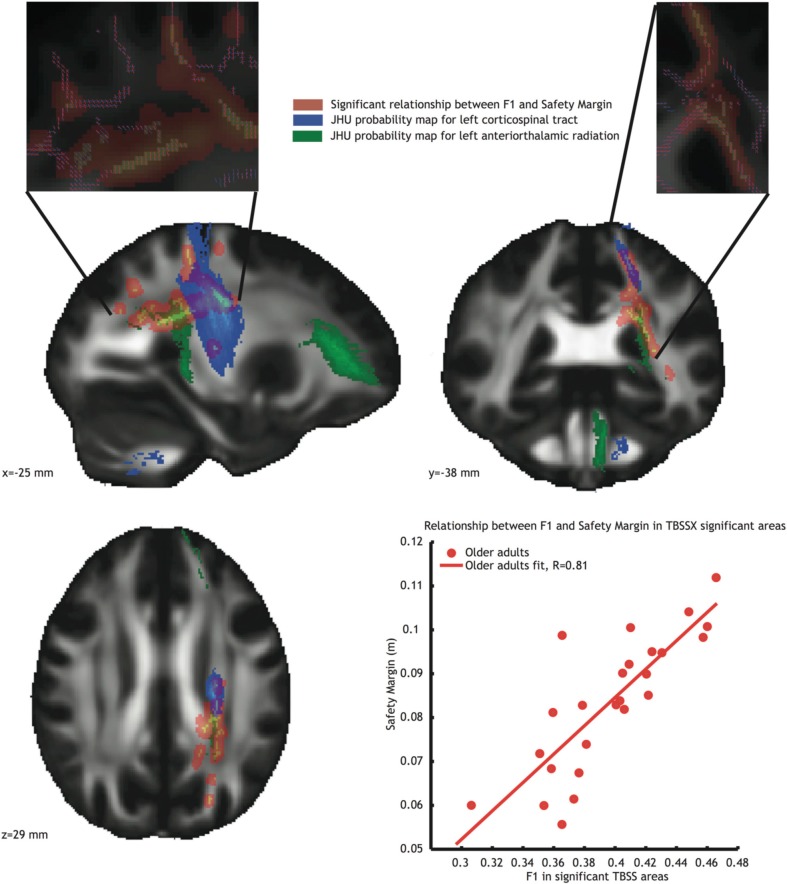
**Voxels showing significant correlations (*P* < 0.05, TFCE corrected) between *FA* in partial volume F1 and safety margin (Orange) in the older adults group.** Blue voxels represent the JHU probability map of the left corticospinal tract, green voxels represent the JHU probability map of the left anterior thalamic radiation. Inlays at the top of the figure show dominant fiber direction in partial volume F1 (red) and F2 (blue). **Bottom right**: Correlation between mean *FA* value in the significant area and safety margin.

Since these regions contain crossing fibers, we also conducted the analysis using TBSSX, which distinguishes between two crossing fiber populations. After doing so, a significant relationship remained for safety margin, which was positively correlated (*r* = 0.81 see Figure 4, orange voxels, and bottom right inlay) with white matter *FA* in the F1 volume. This F1 volume contained mostly vertically oriented volumes (see top inlays in Figure 4, in which the red lines represent the fiber orientation of F1, and the blue lines the fiber orientation of F2), suggesting that the correlations originated from *FA* in the left corticospinal tract (JHU probability map indicated in blue in Figure 4) and left anterior thalamic radiation (JHU probability map indicated in green in Figure 4). The correlation between F1 and safety margin was again significantly higher in the older adults group than in the younger adults group (Fischer r-to-z transform, *P* < 0.01).

## Discussion

We set out to study age related changes in gait stability, white matter microstructural organization, and correlations between these measures. In doing so, we applied more sophisticated measures of gait stability than previous reports. Moreover, specific limitations of the standard tensor model for diffusion were accounted for with the application of a crossing fiber model. In addition to changes in some gait stability parameters, we also found lower white matter *FA* values in the older as compared to young adults group. Significant correlations between behavioral and anatomical measures indicated that older participants with lower white matter *FA* walked with narrower step widths and smaller safety margins.

### Age related changes in gait stability

Similar to previous studies, we found that older adults were less locally stable, i.e., they had a higher λ_*S*_ than younger adults (Kang and Dingwell, 2008, 2009; Cignetti et al., 2011; Toebes et al., 2012). Higher values of λ_*S*_ have been linked to a higher probability of falling (Bruijn et al., 2012; Toebes et al., 2012).

Despite the increase in local instability, we found no evidence for increased age-related instability when assessed with other parameters such as step width and safety margin. Previous reports measuring step width in older adults have reported different results, with some studies finding increased step width (Dean et al., 2007), and others suggesting that age has little effect (Hollman et al., 2011). Increasing step width has been shown to increase the energetic cost of walking (Dean et al., 2007). Thus, in the older adults there may have been a trade-off between widening step width to maintain stability, and keeping step width as narrow as possible to reduce energy consumption. Furthermore, the use of a split-belt treadmill may have led to an increase in step width and safety margin in the younger participants, masking differences between the groups that may be more apparent during level walking.

### Age related changes in white matter microstructural organization

In agreement with previous studies we observed age related *FA* decreases throughout the brain (Nusbaum et al., 2001; Sullivan and Pfefferbaum, 2006, 2007). Such reductions in white matter organization have been shown to be related to performance decreases in a number of functional tasks, of which posture (Van Impe et al., 2012) and gait (Bhadelia et al., 2009) may be closest to our study aim. It should be kept in mind that ageing is not only accompanied by changes in white matter microstructure, but also changes in gray matter volume (Courchesne et al., 2000; Resnick et al., 2003; Giorgio et al., 2010). Nevertheless, here, we studied the relationship between white matter microstructural architecture as such. Below, we will discuss correlations between these metrics of white matter microstructure and gait stability.

### Correlations between white matter microstructural organization and gait stability

In the older adults group, we found significant positive correlations between stability measures (step width and safety margin) and *FA* values in areas located in the anterior thalamic radiation, left longitudinal fasciculus and corticospinal tract. More specifically, older adults with lower *FA* values walked with narrower step widths and smaller safety margins. Since these regions contain crossing fibers, we used a crossing fiber model and TBSSX to further increase the anatomical specificity of our finding. In doing so, we were able to narrow down the positive correlations between safety margin and *FA* values to parts of the left anterior thalamic radiation and left corticospinal tract.

Several previous studies have shown correlations between measures of white matter microstructural organization and gait metrics (Starr et al., 2003; Bhadelia et al., 2009; Srikanth et al., 2010; Zheng et al., 2011; de Laat et al., 2012; Zheng et al., 2012). A recent study by Koo et al. (2012) used the TBSS method to identify relationships between fall risk and white matter metrics in a cohort of 125 homebound older adults. Significant correlations between increased fall risk and reduced *FA* in the medial frontal and parietal subcortical pathways, genu and splenium of corpus callosum, posterior cingulum, prefrontal and orbitofrontal pathways, and frontoparietal and frontotemporal pathways were found. Whereas Koo et al. (2012) assessed fall risk by using the Tinetti scale, our current study extends these findings by showing that *FA* metrics of the left corticospinal tract and thalamic radiation are also specifically correlated with gait stability measures. It is well known that the corticospinal tract is the major pathway for the transmission of neural command signals from the sensorimotor cortex to the spinal cord, and thus, its role in some aspects of gait stability may not be surprising.

The anterior thalamic radiation is an important tract for the connection between the thalamus and frontal cortex. This may indirectly suggest that the frontal structures (and, as a consequence the connections with these frontal structures) are important for gait stability. This seems to be in line with a study by Taubert et al. (2011), who showed increased fronto-parietal resting state network connectivity after two sessions of training a dynamic balancing task, and decreases in white matter metrics in the fronto-parietal region after further training sessions, thus highlighting the importance of these areas for dynamic balancing tasks. Gait stability is different from standing balance, but the fact that a study recording EEG during gait also found activity in the (pre) frontal cortex, suggests that this region may be involved in gait as well (Gwin et al., 2011). Our findings further suggest that its function in gait may be partly related to gait stability.

It is interesting to note that when using the crossing fiber model, only safety margin showed significant positive correlations with white matter microstructural organization in the older adults group, although this measure was also highly correlated with step width. From a control perspective, this seems to make sense as safety margin is more interesting than actual step width when controlling gait in the ML plane. For instance, if trunk motion is large an increased step width may not be sufficient to counteract this effect. On the other hand safety margin takes into account movements of the trunk and foot placement, and would thus be a more useful variable to control.

All significant correlations were found in the left hemisphere. The fact that all our participants were right handed may have contributed to this, as there is abundant evidence that the left hemisphere is dominant for motor control in right-handers (Serrien et al., 2006). Moreover, in a recent EEG study the left sensorimotor cortex was the first area to respond to a loss of balance during walking (Sipp et al., 2012). These results appear to be in line with our finding that the control of gait stability shows a strong left hemispheric lateralization. Studies on posture that provide indications toward lateralization (Laufer et al., 2003; Spinazzola et al., 2003; Ioffe et al., 2010) suggest that it is important to consider the various aspects of balance control. It should be kept in mind that postural control is markedly different from gait stability (Kang and Dingwell, 2006). Our study suggests that the left hemisphere appears to be the crucial one for the control of gait stability in the mediolateral plane by means of foot placements.

### Stabilizing strategies?

Interestingly, we found no correlations between λ_*S*_ (i.e., the rate at which small perturbations are attenuated) and white matter microstructural organization. We did however find strong positive correlations for step width and safety margin in the older adults group, which have been suggested to be the “control variables” of gait (Hof et al., 2007; Hof, 2008). These “control variables” can also be used in a stabilizing strategy when one is unable to attenuate small perturbations (which corresponds to high λ_*S*_ values McAndrew et al., 2010; Hak et al., 2012; McAndrew Young and Dingwell, 2012a,b; McAndrew Young et al., 2012). It could be that due to limitations in white matter integrity, not all older adults with high values of λ_*S*_ were able to increase step width/safety margin, and consequently, a direct one-to-one relationship between λ_*S*_ and safety margin was not found. If indeed altered white matter microstructural organization would limit the ability to increase step width/safety margin in some participants with high values of λ_*S*_, this should show up as a significant interaction effect between *FA* (or F1) and λ_*S*_ when predicting step width/safety margin. In a *post-hoc* analysis, we were unable to show such an interaction effect. Moreover, the fact that older as compared to young adults showed a significantly higher λ_*S*_, but not a significantly wider step width or higher safety margin fails to support this idea.

In the literature, there seems to be little agreement on the idea that an increased step width represents a compensatory strategy. Chamberlin et al. (2005), studying overground walking, reported that increased step width was correlated to fear of falling only (and not the number of falls), which suggests that increased step width may be a compensatory strategy. However, Maki (1997), also studying overground walking, found that an increased step width was also a predictor for falls, thus refuting the idea of step width as a compensatory strategy. However, all of these studies only examined step width, and not safety margin (which may be of more importance, because it takes into account the movements of the CoM). At present, the question of whether increases in step width are used as “stabilizing strategy” in older adults remains to be answered.

### Limitations of the current study

Since we looked for parameters related to fall risk and since we did not focus on fall history, we are unable to conclude whether the presently studied older adults actually experienced falls. Nevertheless, even when our older adults may not actually fall more frequently, given the fact that they have a higher maximum Lyapunov exponent, there is increasing evidence that they are at least more prone to falls (Bruijn et al., 2013).

One further limitation of the present study is that, even though older adults suffering from diseases such as diabetes or neurological insults were excluded from our study, there was no systematic evaluation of sensory deficits, known to be present in elderly (Goble et al., 2009). Such deficits may have contributed to some of the findings on gait instability in this group. Hence, it remains for further study to disentangle age and neuropathy.

## Conclusion

In our group of older adults we found that a decline in white matter microstructural organization strongly predicted lower gait stability. The fact that these correlations were found in the anterior thalamic radiation (a structure connecting frontal cortex and thalamus), may indirectly suggest a role for prefrontal involvement in gait stability in the frontal plane. This is consistent with increased cognitive control of gait stability in older adults. The specific involvement of the left corticospinal tract underlines the possibility of lateralization of stability control during gait.

### Conflict of interest statement

The authors declare that the research was conducted in the absence of any commercial or financial relationships that could be construed as a potential conflict of interest.

## Supplementary material

Supplementary Figure 1 **Calculation of the maximum Lyapunov exponent. (A)** A 3 dimensional attractor (note that analysis were performed on a 5-dimensional attractor, which cannot be visualized) created from the pelvis velocity time series (*q*), and copies delayed by τ and 2 τ. **(B)** Close-up view of part of the attractor. For each point *q*(*j*) on the attractor, the nearest neighbor was calculated and divergence of these points was calculated as *d*_*j*_(*t*). **(C)** The maximum Lyapunov exponents (λ_*S*_) can be calculated as the slope of the average logarithmic rate of divergence curve at 0–0.5 strides.
