# Supplementary material for: White matter microstructural organization and gait stability in older adults
Source: Front Aging Neurosci. 2014 Jun 10;6:104. doi: 10.3389/fnagi.2014.00104 (PMC4051125; doi:10.3389/fnagi.2014.00104)
Supplement: Supplementary file 1 [file DataSheet1.DOCX]

# *Supplementary materials*

# Calculation of stability measures

All stability measures used have been described in great detail in a recent review ([Bruijn, et al., 2013](#_ENREF_8)). In this review, the validity of these measures was also shown to be good.

# The extrapolated centre of mass concept.

In simple mechanical systems, stability can be defined in a straightforward manner. An example of such a system is the inverted pendulum, which is often used as a model for human standing. In this model the center of mass (**CoM**) needs to be controlled such that it is maintained over the base of support (BoS). This simple model holds only for static conditions. For example, when the CoM is outside the BoS but moving towards it, the pendulum may still be stable. Because walking is not static this simple model does not hold true, and needs to be extended taking into account the velocity of the **CoM** and BoS.

For the full derivation of the extended inverted pendulum model, we refer to ([Hof, et al., 2005](#_ENREF_36)). Here, we confine ourselves to a description of how the method may be applied to human gait.

For the calculation of the margin of stability and the temporal stability margin, first the position of the whole body **CoM** and BoS need to be known. Next, the **XCoM** is calculated as:

$\boldsymbol{XCoM}=\boldsymbol{CoM}+\frac{\boldsymbol{V}_{CoM}}{\omega_{0}}$

with **V**_CoM_ being the **CoM** velocity and ω_0_ being the inverted pendulum’s eigenfrequency:

$\omega_{0}=\sqrt{\frac{g}{l}}$

where g represents the acceleration of gravity (9.81 m/s^2^) and l is equivalent to the pendulum length of the participant. The margin of stability is defined as:

$\boldsymbol{b}=\boldsymbol{BoS}-\boldsymbol{XCoM}$

This margin was calculated in the ML direction, during single stance, and the ML centre of pressure position was taken as BoS.

# Maximum Lyapunov exponent

The maximum Lyapunov exponent (λ_s_) quantifies the average logarithmic rate of divergence of a system after a small perturbation ([Dingwell and Cusumano, 2000](#_ENREF_17), [Rosenstein, et al., 1993](#_ENREF_65)). The general idea is that if a system is (or was) at nearly the same state as the current state (i.e., same position, velocity, acceleration, jerk, etc.) either state may be regarded as a perturbation of the other. If we now follow the distance between these two states in time we may find that this distance increases, in which case the system is locally unstable (since a small perturbation could lead to a different behavior), or we may find that it decreases, in which case we would call the system locally stable.

The great advantage of this measure is that it may be calculated from any source of kinematic data, regardless of the reference frame in which the data are recorded ([Bruijn, et al., 2010](#_ENREF_9), [Gates and Dingwell, 2009](#_ENREF_24)).

To reduce the effects of non-stationarity (i.e. wandering around on the treadmill ([Dingwell and Cusumano, 2000](#_ENREF_17))), we calculated λ_S_ from the mediolateral velocity time series of the pelvis cluster. The first 150 strides of the time series were resampled so that on average each stride was 100 samples in length ([Bruijn, et al., 2009](#_ENREF_10), [England and Granata, 2007](#_ENREF_21), [Gates and Dingwell, 2009](#_ENREF_24)). Next a 5-dimensional state space was created from the resampled velocity time series, 4 time delayed copies (i.e. velocity time series and 10, 20 30 and 40 samples delayed copies of the velocity time series, see ([Bruijn, et al., 2013](#_ENREF_8))). For each data point in state space the nearest neighbour was identified, and the Euclidian distance between the two trajectories originating from these points was followed over time. The mean of the log of all values on the time-distance curves was then calculated. The slope of the resulting "divergence curve" (λ_S_) was calculated for the segment from 0-0.5 strides (see also supplementary figure 1)
