# Supplementary material for: White matter microstructural organization and gait stability in older adults
Source: Front Aging Neurosci. 2014 Jun 10;6:104. doi: 10.3389/fnagi.2014.00104 (PMC4051125; doi:10.3389/fnagi.2014.00104)

Supplementary figure 1

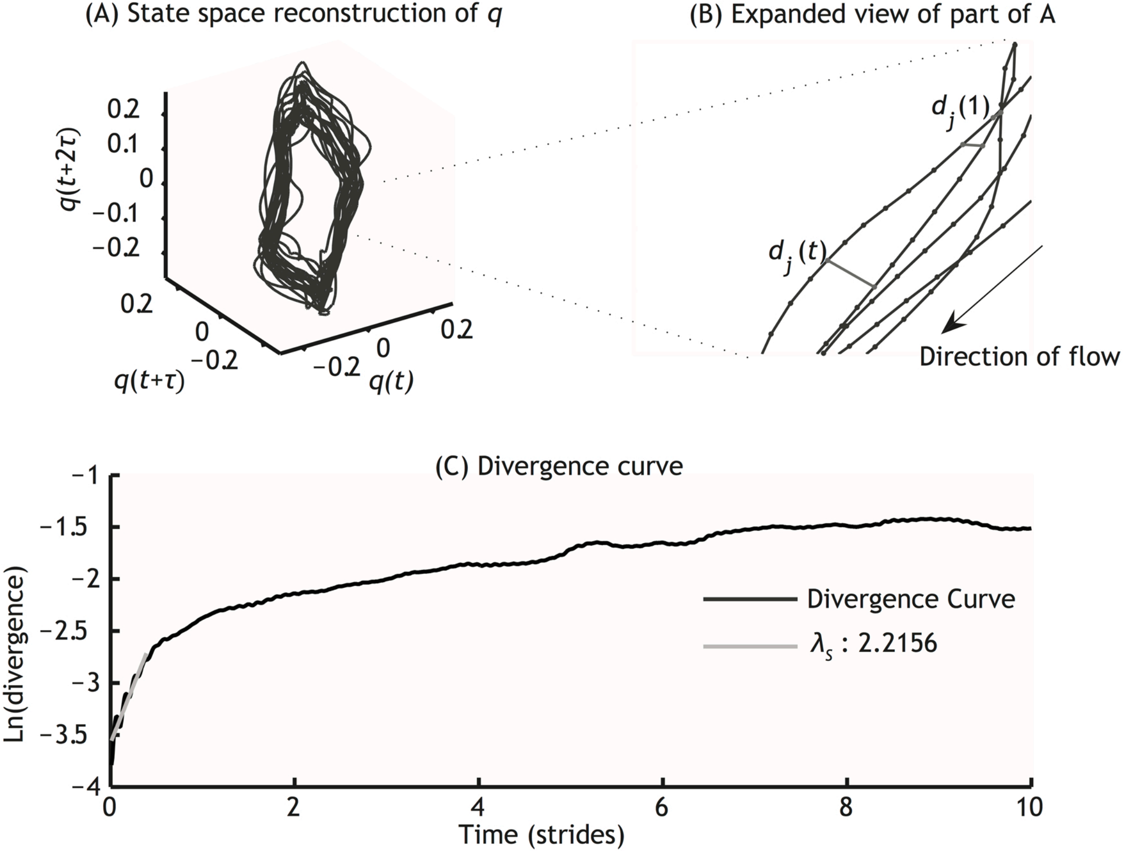

Supplement: Supplementary Figure 1 — Calculation of the maximum Lyapunov exponent. (A) A 3 dimensional attractor (note that analysis were performed on a 5-dimensional attractor, which cannot be visualized) created from the pelvis velocity time series (q), and copies delayed by τ and 2 τ. (B) Close-up view of part of the attractor. For each point q(j) on the attractor, the nearest neighbor was calculated and divergence of these points was calculated as dj(t). (C) The maximum Lyapunov exponents (λS) can be calculated as the slope of the average logarithmic rate of divergence curve at 0–0.5 strides. [file Presentation1.PDF]
